# Motivations Toward Using Digital Health and Exploring the Possibility of Using Digital Health for Mental Health in Bangladesh University Students: Cross-sectional Questionnaire Study

**DOI:** 10.2196/34901

**Published:** 2022-03-04

**Authors:** Munjireen S Sifat, Sandra L Saperstein, Naima Tasnim, Kerry M Green

**Affiliations:** 1 TSET Health Promotion Research Center Stephenson Cancer Center University of Oklahoma Oklahoma City, OK United States; 2 Department of Behavioral and Community Health School of Public Health University of Maryland College Park, MD United States; 3 BRAC University Dhaka Bangladesh

**Keywords:** digital health, mental health, Bangladesh, university students, mental health service use, mobile phone

## Abstract

**Background:**

Digital health is efficacious for the management and prevention of mental health (MH) problems. It is particularly helpful for the young adult population, who appreciate the autonomy digital health provides, and in low-income countries, where the prevalence of MH problems is high but the supply of professionals trained in MH is low.

**Objective:**

The objectives of this study are 2-fold: to determine whether university students in Bangladesh find using digital health for MH promotion acceptable and to examine motivational factors for using digital health for MH.

**Methods:**

This study used a cross-sectional survey to examine the likelihood that university students in Bangladesh (n=311) would use different forms of digital health platforms for MH promotion and assessed drivers of intention to use and actual use of digital health generally and digital health for MH through the lens of the Technology Acceptance Model. The results provided evidence that the university student population in Bangladesh is likely to use digital health to promote their MH.

**Results:**

Social influence (adjusted odds ratio [aOR] 1.68, 95% CI 1.40-2.01; *P*<.001), ease of use (aOR 1.85, 95% CI 1.35-2.53; *P*<.001), and perceived usefulness (aOR 4.12, 95% CI 1.79-9.51; *P*=.001) of digital health were found to be significant drivers of the intention to use general digital health, and having an intention to use digital health (aOR 2.10, 95% CI 1.17-3.78; *P*=.01) had the greatest influence on actual use of digital health. Social influence (aOR 1.71, 95% CI 1.43-2.04; *P*<.001), perceived usefulness (aOR 8.92, 95% CI 4.18-19.04; *P*<.001), and use of general digital health (aOR 2.16, 95% CI 1.18-3.97; *P*=.01) were associated with higher intention to use digital health for MH. The use of general digital health (aOR 4.19, 95% CI 2.37-7.41; *P*<.001) was associated with the actual use of digital health for MH, as were greater non–stigma-related barriers to using traditional clinical MH services (aOR 2.05, 95% CI 1.10-3.80; *P*=.02).

**Conclusions:**

Overall, we see that the use of digital health for MH is acceptable in this population and can be helpful for students who perceive barriers to receiving traditional care. We also gain insight into how to promote the intention to use digital health, which in turn promotes the actual use of digital health.

## Introduction

### Background

Digital health interventions have become widespread to fulfill the need for mental health (MH) services that are low in supply and high in demand [1]. Mobile health (mHealth) is a category within digital health defined as the use of mobile computing and communication technologies in health care and public health [1]. The most common uses of mHealth are apps for monitoring and treating chronic conditions as well as in prevention efforts [2]. mHealth interventions have been found to be beneficial for smoking cessation, adherence to care, health behavior changes, disease management, increasing physical activity [3], and attendance rates of care [4,5]. Marcolino et al [2] examined 23 systematic reviews encompassing >10,000 articles published from 2009 to 2016 and concluded that there is strong evidence to suggest that mHealth is effective in disease management, symptom improvement, and increasing the quality of life of populations.

MH is another domain in which the use of apps has shown promising results. Apps are defined as discrete and independent software that runs on a mobile device [6,7]. Mobile apps have more benefits than SMS text messaging as they can be more deeply personalized [8], visually engage the user, track progress, and be self-paced [9,10]. These features make apps an invaluable platform for the dissemination of interventions. A systematic review evaluated 5646 abstracts published between 2008 and 2013 and found 8 papers describing 5 apps targeting depression, anxiety, and substance abuse that met their inclusion criteria [11]. The review only included evidence-based MH apps that could be downloaded from app stores. The results showed significant reductions in depression, stress, and substance use [11]. Other meta-analyses support that psychological intervention content delivered through a web or mobile app can be as efficacious as face-to-face treatment for depression [12-14].

Digital health can increase the likelihood that health interventions will be delivered to otherwise hard-to-reach populations, particularly in low- and middle-income settings [2]. A systematic review assessed 6 interventions that were specific to low- and middle-income countries and found that 5 out of 6 showed benefits to participants [15]. Other advantages of digital health are convenience, ease, cost-effectiveness, scalability, personalization, and “the ability to send time-sensitive messages with an ‘always on’ device” [16]. Furthermore, it can reach populations who would otherwise not engage with traditional health services [17].

There are particular benefits for governments of low-income countries that need additional support for patient management [18] because digital health is potentially highly accessible in low-income countries, with 60% of low-income populations having access to a mobile phone [19]. Furthermore, internet and smartphone use are rising worldwide in high- and low-income countries alike [20]. Bangladesh is one such low-income country that has shown positive results in the use of digital health for promoting health care in Bangladesh for various health-related issues [21,22]. The Bangladesh government fosters digital development, and the United Nations recognized its efforts toward building a digital health infrastructure in 2011 [23]; as of the beginning of 2020, >99 million people use the internet in Bangladesh [24], and most of them own smartphones [25]. Although the focus of most interventions in Bangladesh has been on the use of SMS text messaging and landlines [21,22], a handful of studies have examined using apps on smartphones for health [22]. In Bangladesh, apps have been used to link village physicians to formal physicians [26] and for diabetes management [27], nutrition services [28], and maternal and child health [29]. A systematic review examined all health-related apps in Bangladesh (N=234), and a total of nine categories of apps were mentioned in the report: general health information apps, physician information apps, institutional apps, fitness apps, mother and child apps, disease-specific care apps, herbal apps, and food and nutrition apps [20]. As such, we see a large number of mobile phone apps being used for health promotion in Bangladesh; yet, none are focused on MH promotion.

Although apps show promise in Bangladesh for other health outcomes, there is a lack of literature examining the use of mHealth for MH in this population or rates of mHealth use in general. This is particularly important given the high rates of MH problems in the population and the current lack of infrastructure in Bangladesh to deal with these problems [30]. According to the World Health Organization, there is <1 (0.001%) psychiatrist available for every 100,000 people in Bangladesh [31,32]. Although there is no national surveillance system that indicates a nationally representative prevalence rate of MH disorders in Bangladesh, a systematic review estimated the prevalence of MH disorders to be between 6.5% and 31% among adults [33]. Another systematic review examining rates of suicide estimated the rate to be 39.6 per 100,000, which is triple the global rate (10.7 per 100,000) [30].

The onset of depression typically occurs from adolescence to early adulthood [34,35]. In particular, early adulthood is deemed the “most vulnerable time” for the onset of depressive symptoms in Bangladesh [30]. This time frame, along with the multiple stressors (academic pressure and new social and physical environments) that college students face, makes the university student population particularly prone to depressive symptoms [36]. Recent studies examined MH outcomes in Bangladesh university students and found high rates of depression, ranging from 47.5% [37] to 69.5% [25]. Evidence supports that, the earlier one can manage stress and depressive symptoms, the better the overall health outcomes they will have [38]. MH apps are particularly well-suited for young adults seeking help for their symptoms because this population reports a high need for autonomy [39,40]. Young adults prefer using self-help materials if they are familiar with the medium that delivers them, such as smartphones [41].

Although the rates of MH problems may be high, there is low MH literacy [33] and high stigma surrounding the topic [28]. Hossain et al [33] found that there was low awareness of MH disorders and that attitudes toward seeking help for MH were negative. They found that even those who had an MH disorder did not prioritize MH care. This is not uncommon in low-income countries in Asia [31,42], where it is believed that MH problems are caused by religious or cultural abnormalities [43], which in turn is associated with low use of clinical services. As mHealth has been used successfully in Bangladesh for chronic disease management [20], it is possible that it can also be used to improve MH. At a minimum, the acceptability of using digital health for MH should be determined. Developing MH messaging for in-app delivery for college students in Bangladesh has the potential to reduce, manage, and prevent depression symptomatology.

The Technology Acceptance Model (TAM) is an information technology framework for understanding users’ adoption and use of emerging technologies [44]. The model proposes that a user’s perception of the usefulness (ie, perceived benefits) and ease of use lead to their intent to use the technology and that intention is directly related to actual use. The TAM also posits that perceptions of usefulness and ease of use are influenced by external factors such as social influences [44]. This study uses this framework to assess where a Bangladeshi population falls on the scale of accepting digital health for MH and describing their current digital health use.

### Objectives

This paper aims to (1) describe the likelihood that students will use different forms of digital health platforms for MH promotion; (2) assess the relationship between the perceived ease of use, usefulness, and social influence on the use of digital health and the intention to use and actual use of digital health; and (3) assess the relationship between the perceived ease of use, usefulness, and social influence on the use of digital health for MH and the intention to use and actual use of digital health for MH.

## Methods

### Study Sample

Adult university students across Bangladesh were invited to take an anonymous web-based survey. Students were emailed a flyer invitation by faculty to participate in the study and offered a 1-in-4 chance to win 422 Bangladeshi taka (US $5) for participating in the survey. In addition to faculty recruitment, flyers were posted on university social media pages. The total sample size was 311 complete responses.

### Survey Creation

A total of 5 cognitive interviews were conducted with Bangladeshi university students to develop the survey. As part of the creation of the survey instruments, first, native Bangla speakers reviewed and translated the English survey items (most items were part of previously validated scales, which is explained further in the *Measures* section of this paper) into Bangla. Items with complex translations or items with cultural meanings that differed in Bangla were noted and compiled into a guide for the cognitive interviews. The cognitive interviews asked the participants to explain how they defined MH and their interpretation of the survey items. Items that were culturally inappropriate or that students did not understand were adapted to make it easier for them to understand. For example, the phrase *feeling down* to denote feeling sad or depressed in the question *How often in the past two weeks did you feel down, depressed, or hopeless?* is not used in Bangladesh and was removed from the question. On the basis of the cognitive interviews, the questionnaire was adapted and pilot-tested with 10 participants. The pilot test participants reported no difficulties with the items, and the survey was completed.

### Measures

#### Dependent Variables

##### Overview

Intention to use general digital health (Cronbach α=.88) was assessed by creating a mean score of three items: (1) *I intend to use a digital health service in the future*, (2) *I will always try to use digital health services in my daily life*, and (3) *I plan to continue to use digital health services frequently* [45]. Intention to use digital health for MH (Cronbach α=.89) was assessed similarly using the mean of three items: (1) *I intend to use digital mental health services in the future*, (2) *I will always try to use digital mental healthcare in my daily life*, and (3) *I plan to continue to use digital mental health services frequently*. Items were scored from 1 (do not agree) to 7 (totally agree) and then dichotomized into no or low (1-4.44) and moderate or high (4.45-7) intention. The cutoff was 4.45 because 4 was considered *neither agree nor disagree* and 5 was considered *slightly agree* on the scale.

##### Current Use

The use of digital health for general health was assessed by asking if the following statement—*I use digital health services to better my health (excluding use for mental health) currently*—was true or false. An example was given in the question stem *For example, using an app to track steps, for weight loss, to increase physical activity*. Similarly, digital health for MH was a binary variable as to whether participants used digital health: *I use digital health for mental health currently (for example, following meditation videos).* These questions have been used in previous studies assessing use of digital health for MH in populations in low-income countries [46].

#### Independent Variables

##### Barriers to Using Clinical MH Services

Barriers to seeking MH services were measured using the Barriers to Access to Care Evaluation scale [47]. The scale consists of both stigma-related and nonstigma-related items. The participants responded using a Likert-scale of 1=not at all (indicating this was not a barrier to care) to 4=a lot (indicating a great barrier to care) to the following question: *Have any of these issues ever stopped, delayed or discouraged you from getting, or continuing with, professional care for a mental health problem?* The respondents rated how much of a barrier the provided scenarios were to receiving MH care, example barriers being *Thinking that professional care probably would not help* or *Concern about what people at work might think, say, or do*. Responses to the items were averaged to create the final score (1-4). The nonstigma (attitudinal and instrumental) barriers subscale of the Barriers to Access to Care Evaluation scale included 22 items and had adequate reliability, with a Cronbach α of .76. The stigma subscale consisted of 12 items and had a Cronbach α of .89.

Variables in relation to general digital health and digital health specifically for MH promotion were included.

##### Likelihood of Using Digital Health

This variable was assessed by asking how likely the participants were to (1) text a helpline or crisis center, (2) text a professional (ie, therapist or physician), (3) use a smartphone app for self-paced meditation or nonclinical practices, (4) use a smartphone app to look up information and symptoms about MH, (5) use internet-based self-paced programs for meditation or nonclinical practices, and (6) use internet-based programs to video chat with a professional on a scale of 1 (extremely unlikely) to 5 (extremely likely).

##### Ease of Use of Digital Health

This variable was assessed by averaging the scores of 6 items measured on a 7-point scale (1=do not agree, 7=strongly agree). Example items included *Learning how to use digital health services is easy for me*, *My interaction with digital health service is clear and understandable*, and *I find digital health services easy to use* [45]. The Cronbach α for these items was .89.

##### Social Influence on Digital Health Use

This variable was assessed using the mean of the following three items on a 7-point scale (1=do not agree, 7=strongly agree): (1) *People who are important to me think that I should use a digital health service*, (2) *People who influence my behavior think that I should use a digital health service*, and (3) *People whose opinions that I value prefer that I use digital health service* [45]. The Cronbach α for these items was .94. Social influence regarding the use of digital health for MH was assessed using the mean of 3 items measured on a scale of 1 (do not agree) to 7 (strongly agree); for example, *People who are important to me think that I should use digital mental health services.* The Cronbach α for these items was .95.

##### Perceived Usefulness of General Digital Health

This variable was assessed by taking the mean of two items: *I find digital health services useful in my daily life* and *Using digital health services helps me accomplish things more quickly* [45]. Both were measured on a scale of 1 (do not agree) to 7 (totally agree). Perceived usefulness of digital health for MH was assessed by taking the mean of 3 items and dichotomizing the measure into a scale of 0 (low perceived usefulness; *do not agree* to *neither agree nor disagree*) to 1 (high perceived usefulness; *slightly agree* to *totally agree*). The construct was dichotomized as a method of addressing collinearity between this construct and the ease of use construct. The following is an example item: *I find that digital mental health services are or could be useful in my daily life*. The Cronbach α for these items was .88.

#### Covariates

##### Wellness

This variable was measured using the 5-item HERO Wellness Scale by Yaklin et al [48]. The scale assesses happiness, enthusiasm, resilience, and optimism, and had high-reliability scores in the study sample (Cronbach α=.87). An example item—*On average, during the last seven days, how optimistic have you felt?*—was scored on a scale of 0 (not at all) to 10 (extremely). Final scores were created by summing the answers to all items and ranged from 0 to 50, with higher scores indicating higher wellness.

##### Perceived Stress

This variable was measured using the 4-item Perceived Stress Scale [49] and had an acceptable reliability score in the study sample (Cronbach α=.70). Questions such as *In the last month, how often have you felt that you were unable to control the important things in your life?* were answered on a scale of 0 (never) to 4 (very often) and were summed, with final scores ranging from 0 to 16 and higher scores indicating higher stress.

##### Depression

This variable was assessed using the 2-item (*r*=0.53; *P*<.001) Patient Health Questionnaire [50]. Questions such as *In the past two weeks, how often have you felt depressed or hopeless?* were answered on a scale of 0 (never) to 3 (almost every day). Scores were dichotomized into whether one was likely to have a major depressive disorder based on a cutoff point of 3 from the sum of the scale items.

##### Lifetime Suicidal Ideation

This was assessed as a binary variable (yes or no) as to whether they had ever had thoughts that they would rather be dead.

##### Physical Health

This variable was assessed using one item: *How would you rate your overall health?* (1=poor, 5=excellent).

### Demographics

Socioeconomic status (SES) while growing up was assessed by asking *How often did your family have enough money to make ends meet?* Respondents answered on a scale of 0 (never) to 5 (always), and the answers were dichotomized into low versus high SES. Gender was measured in three categories: male, female, and gender minority. Age was measured as a continuous variable. Relationship status was assessed categorically; the participants selected if they were single, partnered (in a relationship or married), or other. Semester or year in school was categorized as first to third or first year, fourth to sixth or second year, seventh to ninth or third year, 10th to 12th or fourth year, and 13th or fourth year or higher. The degree of study was dichotomized as pursuing either a bachelor’s or master’s degree. Geographic location was assessed by asking if the participants lived in a rural or urban setting.

Specific digital health indicators of interest were payment methods, where responses indicated if the participants had monthly plans, pay as you go, or something else. The participants were also asked about their language preference for digital health and if they preferred their native language (Bangla), English, or something else.

### Analysis

Analyses were conducted using complete case analysis with a final sample size of 311. Means, SDs, and frequencies were used to describe the data. Group differences between the primary outcome of interest—use of digital health for MH—and demographic variables were assessed using analysis of variance and chi-square tests. Logistic regression analysis was used to examine the unadjusted relationships between individual predictors and outcomes of interest. If the unadjusted association was found to be associated at *P*≤.20, the variable was included in a final, adjusted logistic regression model. Models were shown to predict the intention to use and actual use of general digital health and digital health for MH. The models predicting actual use included hierarchical regression, with the first step showing unadjusted associations, the second step showing the model without including intention to use, and the final step (step 3) including the intention to use. Model fit statistics were reported.

### Ethics Approval

The study was approved by the University of Maryland College Park (UMCP) Institutional Review Board (IRB number: 1656046-3)

## Results

### Overview

Descriptive statistics were used to describe the sample demographics in Table 1. Differences between those who used the primary outcome of digital MH and those who did not were examined within demographic variables. The sample was predominantly male (184/311, 59.2%), identified as heterosexual (276/311, 93.9%), not in a relationship (239/311, 76.8%), and sought a bachelor’s degree (258/311, 83%). Growing up, the participants were mostly from families with a high SES (223/311, 71.7%) and from urban areas (167/311, 53.7%). The only significant differences among the variables of interest between those who used digital health for MH and those who did not were gender and whether they used general digital health. The participants who reported using digital health for MH (82/311, 26.4%) used general digital health at nearly twice the rate (57/82, 70%) of those who did not use digital health for MH (25/82, 30%), a significant difference (*P*<.001). Men were less likely to use digital health for MH than women and gender minorities—of those who did not use digital health for MH, 62.4% (143/229) were men and 37.6% (86/229) were women or gender minorities (*P*=.049).

Students had moderate levels of wellness (mean 26.58, SD 9.94), self-reported health status (mean 2.69, SD 0.87), and perceived stress (mean 8.46, SD 0.87). Approximately 43.4% (135/311) of the sample were likely to have depression, and 28% (78/311) reported lifetime suicidal ideation. Most students used a monthly plan to pay for their phones (223/311, 71.7%), owned their phones (308/311, 99%), and used a smartphone (310/311, 99.7%). In the sample, 43.4% (135/311) reported using digital health for general health, and 26.4% (82/311) used digital health for MH. Although half of the sample (115/311, 49.8%) did not have a preference between their native language (Bangla) and English, 31.5% (98/311) preferred Bangla and 18.6% (58/311) preferred English.

**Figure 1:**
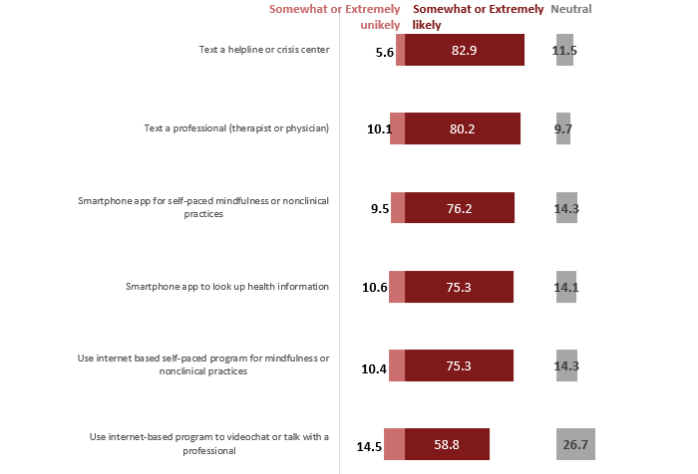
Distribution of likelihood of using digital health forms for mental health promotion (%; N=311). Somewhat likely and extremely likely were combined in the likely category. Somewhat unlikely and extremely unlikely were combined in the unlikely category.

Respondents reported their likelihood of using different forms of digital health for MH promotion (Figure 1). Overall, a large percentage (227/302, 75.3% to 246/297, 82.9%) of the sample reported likelihood of using apps and internet-based programs. Most respondents said they would be likely to text a helpline or crisis center (170/290, 58.8%) or a professional (ie, therapist or physician; 220/292, 75.3%), use an app on a smartphone for self-paced meditation or nonclinical practices (227/302, 75.3%), look up information and symptoms about MH (229/301, 76.2%), use internet-based self-paced programs for meditation or nonclinical practices (242/302, 80.2%), or talk with a professional (250/301, 82.9%).

A correlation matrix of the independent variables used in all models is shown in Table 2. When examining the main constructs of the TAM related to general digital health, we found significant correlations between social influence and ease of use of general digital health (*r*=0.316; *P*<.001) and perceived usefulness (*r*=0.241; *P*<.001). For the variables related to digital health for MH, there were significant correlations among the ease of use of digital health construct, social influence (*r*=0.256; *P*<.001), and perceived usefulness (*r*=0.366; *P*<.001). There were also significant correlations among the control variables of interest—wellness was negatively correlated with stress (*r*=−0.560; *P*<.001) and depression (*r*=0.338; *P*<.001). Geography and SES were correlated (*r*=0.190; *P*<.001) in that those who lived in urban areas had higher SES. Perceived general health was positively correlated with wellness (*r*=0.382; *P*<.001) and negatively correlated with stress (*r*=−0.292; *P*<.001). Perceived stigma as a barrier to MH care and instrumental and attitudinal barriers to care were highly correlated (*r*=0.765; *P*<.001).

**Table 1:** Participant demographics (N=311).

Demographics			Overall		Did not use digital health for MH^a^ (n=229)		Used digital health for MH (n=82)		Chi-square *P* value or ANOVA^b^ *P* value	
Age (18-41 years), mean (SD)			22.7 (1.86)		22.8 (1.74)		22.6 (2.18)		.59	
**Gender, n (%)**										.049
	Male	184 (59.2)		143 (62.4)		41 (50)				
	Female and gender minority	127 (40.8)		86 (37.6)		41 (50)				
**Sexual orientation, n (%)**										.69
	Heterosexual or straight	276 (93.9)		203 (93.5)		73 (94.8)				
	Sexual minority (LGBTQA+^c^)	18 (6.1)		14 (6.5)		4 (5.2)				
**Childhood SES^d^** **, n (%)**										.73
	Low	88 (28.3)		66 (28.8)		22 (26.8)				
	High	223 (71.7)		163 (71.2)		60 (73.2)				
**Relationship status**, n (%)										.65^e^
	Single	239 (76.8)		179 (78.2)		60 (73.2)				
	Partnered (relationship or married)	69 (22.2)		48 (21.0)		21 (25.6)				
	Other (self-described)	3 (1.0)		2 (0.9)		1 (1.2)				
**Semester or year in school, n (%)**										.09
	First to third or first year	64 (20.6)		51 (22.4)		13 (15.9)				
	Fourth to sixth or second year	60 (19.4)		38 (16.7)		22 (26.8)				
	Seventh to ninth or third year	62 (20.0)		43 (18.9)		19 (23.3)				
	10th to 12th or fourth year	60 (19.4)		43 (18.9)		19 (23.2)				
	13th or fourth year or higher	64 (20.6)		53 (23.3)		11 (13.4)				
**Degree of study, n (%)**										.17
	Bachelor’s (BS^f^ or BA^g^)	258 (83.0)		186 (81.2)		72 (87.8)				
	Master’s (MPH^h^ or MBA^i^)	53 (17.0)		43 (18.8)		10 (12.2)				
**Geographic location, n (%)**										.30
	Rural	144 (46.3)		102 (44.5)		42 (51.2)				
	Urban	167 (53.7)		127 (55.5)		40 (48.8)				
Wellness (0-50), mean (SD)			26.6 (9.94)		26.2 (9.73)		27.5 (10.48)		.32	
Perceived stress (0-16), mean (SD)			8.46 (3.42)		8.42 (3.48)		8.53 (3.24)		.81	
High depressive symptoms (>3), n (%)			135 (43.4)		104 (45.4)		31 (37.8)		.23	
Suicidal ideation (lifetime), n (%)			78 (28.0)		58 (28.4)		20 (26.7)		.77	
Rating of health status (1-5), mean (SD)			2.69 (0.87)		2.69 (0.89)		2.68 (0.86)		.92	
**Mobile phone plan, n (%)**										.98
	Monthly plan	223 (71.7)		164 (71.6)		59 (72)				
	Pay as you go	52 (16.7)		38 (16.6)		14 (17.1)				
	Other	36 (11.6)		27 (11.8)		9 (11)				
**Phone ownership, n (%)**										.78
	Personal phone	308 (99.0)		227 (99.1)		81 (98.8)				
	Shared phone	3 (1.0)		2 (0.9)		1 (1.2)				
**Type of phone, n (%)**										.55
	Phone with internet capability	310 (99.7)		228 (99.6)		82 (100)				
	Phone without internet capability	1 (0.3)		1 (0.4)		0 (0)				
General digital health use, n (%)			135 (43.4)		78 (34.1)		57 (69.5)		<.001	
Use of digital health for MH, n (%)			(82) 26.4		N/A^j^		N/A		N/A	
**Language preference for digital health, n (%)**										.36
	Bangla	98 (31.5)		77 (33.6)		21 (25.6)				
	English	58 (18.6)		40 (17.5)		18 (22.5)				
	Bangla or English	115 (49.8)		112 (48.9)		43 (52.4)				

^a^MH: mental health.

^b^ANOVA: analysis of variance.

^c^LGBTQA+: lesbian, gay, bisexual, transgender, queer, asexual plus other identities.

^d^SES: socioeconomic status. Item asked How often did your family have enough money to make ends meet growing up? Low=never, rarely, sometimes; high=most of the time, always.

^e^This chi-square test is not valid as n<5 for some cells.

^f^BS: bachelor of science.

^g^BA: bachelor of arts.

^h^MPH: master of public health.

^i^MBA: master of business administration.

^j^N/A: not applicable.

**Table 2:** Correlation matrix (N=311)^a^.

	1^b^	2^c^	3^d^	4^e^	5^f^	6^g^	7^h^	8^i^	9^j^	10^k^	11^l^	12^m^	13^n^	14^o^
1	—^p^	.32^q^	.24^q^	.80^q^	.30^q^	–.05	.10	–.06	−.19^q^	.05	−.10	−.06	.03	.01
2	—	—	.37^q^	.26^q^	.37^q^	.10	.14^r^	−.11	−.05	.16^q^	.11	−.13^r^	−.21^q^	−.22^q^
3	—	—	—	.19^q^	.44^q^	.02	.08	−.06	−.09	.02	−.04	.01	−.14^r^	−.11
4	—	—	—	—	.33^q^	−.03	.07	−.04	−.21^q^	.01	−.06	−.10	.09	.05
5	—	—	—	—	—	.10	.04	−.01	−.10	−.05	.02	.05	−.02	.01
6	—	—	—	—	—	—	.38^q^	−.29^q^	−.17^q^	.06	.15^r^	−.22^q^	−.19^q^	−.18^q^
7	—	—	—	—	—	—	—	−.56^q^	−.19^q^	−.02	.08	−.34^q^	−.^25q^	−.25^q^
8	—	—	—	—	—	—	—	—	.17^q^	.01	.00	.45^q^	.26^q^	.24^q^
9	—	—	—	—	—	—	—	—	—	.14^r^	.10	−.03	.03	.06
10	—	—	—	—	—	—	—	—	—	—	.19^q^	−.10	−.12^r^	−.09
11	—	—	—	—	—	—	—	—	—	—	—	−.06	−.17^q^	−.20^q^
12	—	—	—	—	—	—	—	—	—	—	—	—	.25^q^	.26^q^
13	—	—	—	—	—	—	—	—	—	—	—	—	—	.76^q^
14	—	—	—	—	—	—	—	—	—	—	—	—	—	—
Value, mean (SD)	4.15 (1.84)	5.35 (1.16)	5.37 (1.20)	4.10 (1.82)	5.15 (1.18)	2.69 (0.88)	26.6 (9.94)	8.46 (3.42)	0.41 (0.49)	1.54 (0.50)	0.72 (0.45)	0.43 (0.50)	0.70 (0.64)	0.87 (0.46)

^a^Higher scores equal greater amounts for all variables.

^b^Social influence on the use of general digital health; range: 1-7.

^c^Ease of use of general digital health; range: *poor* to *excellent*.

^d^Perceived usefulness of general digital health (0=low, 1=high).

^e^Social influence on the use of digital health for mental health (0=low, 1=high); range: 1-7.

^f^Perceived usefulness of digital health for mental health (0=low, 1=high).

^g^General health rating; range:0-50.

^h^Wellness; range: 0-50.

^i^Perceived stress; range: 0-16.

^j^Gender (0=male, 1=female).

^k^Geography (0=rural, 1=urban).

^l^Socioeconomic status (0=low, 1=high).

^m^Depression (0=low, 1=high).

^n^Stigma-related barriers to care; range: 1-4.

^o^Attitudinal and instrumental barriers to care.

^p^Not applicable.

^q^*P*<.001.

^r^*P*<.01.

### Regression Analysis Results

The outcomes of intention to use and actual use of digital health in general and digital health for MH were examined using logistic regression analysis. Table 3 shows the results for the outcome of intention to use general digital health. In the unadjusted results, all the main constructs of the TAM (ease of use, social influence, and perceived usefulness of digital health) were positively associated with intention to use digital health, as was perceived wellness. In the adjusted model, these constructs remained statistically significantly associated. Those who perceived digital health to be easy to use (adjusted odds ratio [aOR] 1.85; *P*<.001), had higher approval from their social networks to use digital health (aOR 1.68; *P*<.001), and perceived higher usefulness of digital health (aOR 4.12; *P*=.001) had higher adjusted odds of intending to use digital health. The *R*^2^ for this final adjusted model was 0.445 (*P*<.001).

**Table 3:** Logistic regression analysis associating the Technology Acceptance Model constructs with intention (high vs low) to use digital health (N=311)^a^.

Item		Unadjusted associations			Adjusted model^b,c^	
		OR^d^ (95% CI)	*P* value	aOR^e^ (95% CI)		*P* value
Ease of use of digital health (1-7)^f^		2.29 (1.79-2.93)	<.001	1.85 (1.35-2.53)		<.001
Social influence on digital health use (1-7)^f^		1.79 (1.54-2.09)	<.001	1.68 (1.40-2.01)		<.001
Perceived usefulness of digital health (high vs low)		9.76 (4.70-20.35)	<.001	4.12 (1.79-9.51)		.001
**Controls**						
	Rating of general health (poor to excellent)^f^	1.23 (0.94-1.61)	.14	1.38 (0.95-2.02)		.09
	Wellness (0-50)^f^	1.02 (1.00-1.05)	.04	1.00 (0.97-1.04)		.85
	Perceived stress (0-16)^f^	0.97 (0.91-1.04)	.45	N/A^g^		N/A
	SES^h^ growing up (high vs low)	0.67 (0.39-1.13)	.13	0.56 (0.27-1.16)		.12

^a^Geography (urban vs rural) and gender (female vs male) variables were not significant in the unadjusted model, so they were not included in the adjusted model.

^b^Nagelkerke *R*^2^=0.445.

^c^*P* value <.001

^d^OR: odds ratio.

^e^aOR: adjusted odds ratio.

^f^Higher scores equal greater amounts.

^g^N/A: not applicable.

^h^SES: socioeconomic status.

When examining the outcome of use of digital health, the only TAM construct significantly associated was social influence, as seen in Table 4. However, when the intention to use digital health construct was added to the model (step 3 of the hierarchical regression), social influence was no longer significantly associated with the use of digital health. In the unadjusted analyses shown in step 1 of Table 4, the controls of better health, wellness, and higher SES were associated with actual use; in the adjusted model, only SES remained associated. In the final step of the model, we saw that those with higher intention to use digital health had higher adjusted odds of actual use (aOR 2.10; *P*=.01). The *R*^2^ for this final adjusted model was 0.108 (*P*<.001).

In Table 5, for the analysis looking at predictors of intention to use digital health for MH, we found that social influence, perceived usefulness, and use of general digital health were positively associated with the intention to use digital health for MH in the unadjusted model. In the adjusted model, these constructs remained significantly associated. Higher social influence (aOR 1.73; *P*<.001), perceived usefulness (aOR 8.70; *P*<.001), and use of general digital health (aOR 2.16; *P*=.01) were associated with higher adjusted odds of intention to use digital health for MH. The *R*^2^ for this final adjusted model was 0.492 (*P*<.001).

The results in Table 6 show that social influence, intention to use, use of general digital health, instrumental and attitudinal barriers, and gender were positively associated with using digital health for MH. There were no significant changes when comparing step 2 of the model (without intention to use) and step 3. In the final adjusted model, we see that those who used digital health for their general health had higher odds (aOR 4.19; *P*<.001) of using digital health for MH. Those who perceived higher instrumental and attitudinal barriers to receiving clinical MH care had higher adjusted odds of using digital health for MH (aOR 2.05; *P*=.02), and women had almost twice higher adjusted odds of use when compared with men (aOR 1.88; *P*=.03). These model statistics show that the model is statistically significant (*P*<.001), with an *R*^2^ of 0.204.

**Table 4:** Logistic regression associating the Technology Acceptance Model constructs with use of digital health (N=311)^a^.

Item			Step 1				Step 2^b^				Step 3^c^		
			OR^d^ (95% CI)		*P* value		aOR^e^ (95% CI)		*P* value		aOR (95% CI)		*P* value
Ease of use of digital health (1-7)			1.21 (0.99-1.47)		.06		1.07 (0.86-1.33)		.56		0.97 (0.77-1.23)		.81
Social influence on digital health use (1-7)			1.14 (1.01-1.30)		.03		1.15 (1.01-1.32)		.04		1.08 (0.93-1.25)		.32
Perceived usefulness of digital health (high vs low)			1.39 (0.74-2.61)		.30		N/A^f^		N/A		N/A		N/A
Intention to use digital health (high vs low)			2.29 (1.41-3.72)		.001		N/A		N/A		2.10 (1.17-3.78)		.01
**Controls**													
	Rating of general health (poor to excellent)	1.49 (1.14-1.94)		.004		1.37 (1.03-1.85)		.03		1.34 (0.99-1.80)		.05	
	Wellness (0-50)	1.03 (1.01-1.05)		.01		1.02 (0.99-1.05)		.32		1.01 (0.98-1.05)		.36	
	Perceived stress (0-16)	0.95 (0.89-1.02)		.15		1.00 (0.92-1.09)		.94		1.00 (0.92-1.08)		.93	
	SES^g^ growing up (high vs low)	1.72 (1.03-2.87)		.04		1.64 (0.96-2.82)		.07		1.81 (1.04-3.12)		.04	

^a^Higher scores equal greater amounts. Geography (urban vs rural) and gender (female vs male) variables were not significant in the unadjusted model; therefore, they were not included in the adjusted model.

^b^Nagelkerke *R*^2^=0.08; *P*=.11.

^c^Nagelkerke *R*^2^=0.08; *P*<.001.

^d^OR: odds ratio.

^e^aOR: adjusted odds ratio.

^f^N/A: not applicable.

^g^SES: socioeconomic status.

**Table 5:** Logistic regression associating the Technology Acceptance Model constructs with intention to use digital health for mental health (N=311)^a^.

Item		Unadjusted associations		Adjusted model^b,c^	
		OR^d^ (95% CI)	*P* value	aOR^e^ (95% CI)	*P* value
Ease of use of digital health (1-7)		1.79 (1.44-2.23)	<.001	1.39 (0.99-1.73)	.06
Social influence on the use of digital health for mental health (1-7)		1.89 (1.61-2.21)	<.001	1.71 (1.43-2.04)	<.001
Perceived usefulness of digital health for mental health (high vs low)		15.24 (7.69-30.20)	<.001	8.92 (4.18-19.04)	<.001
Use of general digital health (yes vs no)		2.33 (1.45-3.76)	.001	2.16 (1.18-3.97)	.01
**Barriers to seeking traditional clinical mental health services**					
	Stigma-related barriers (1-4)	0.98 (0.69-1.40)	.91	N/A^f^	N/A
	Instrumental or attitudinal barriers (1-4)	1.00 (0.61-1.65)	.99	N/A	N/A
**Controls**					
	Mental health need (need help vs not)	0.99 (0.63-1.56)	.96	N/A	N/A
	Wellness (0-50)	1.02 (0.99-1.04)	.19	N/A	N/A
	Perceived stress (0-16)	0.98 (0.91-1.04)	.47	N/A	N/A
	SES^g^ growing up (high vs low)	0.74 (0.44-1.23)	.25	N/A	N/A
	Urban vs rural	0.97 (0.62-1.54)	.91	N/A	N/A
	Female vs male	0.67 (0.42-1.06)	.09	1.12 (0.60-2.08)	.73

^a^Higher scores equal greater amounts.

^b^ Nagelkerke *R*^2^=0.49.

^c^*P*<.001.

^d^OR: odds ratio.

^e^aOR: adjusted odds ratio.

^f^N/A: not applicable.

^g^SES: socioeconomic status.

**Table 6:** Logistic regression associating the Technology Acceptance Model constructs with use of digital health for mental health (N=311)^a^.

Item		Step 1		Step 2^b^		Step 3^c^	
		OR^d^ (95% CI)	*P* value	aOR^e^ (95% CI)	*P* value	aOR (95% CI)	*P* value
Ease of use of digital health (1-7)		1.20 (0.95-1.51)	.12	1.12 (0.86-1.48)	.40	1.11 (0.84-1.47)	.45
Social influence on the use of digital health for mental health (1-7)		1.18 (1.02-1.37)	.02	1.14 (0.96-1.36)	.12	1.11 (0.92-1.34)	.26
Perceived usefulness of digital health for mental health (high vs low)		1.93 (1.00-3.73)	.05	1.40 (0.65-3.04)	.40	1.26 (0.55-2.89)	.59
Intention to use digital health for mental health (high vs low)		2.29 (1.31-4.01)	.004	N/A^f^	N/A	1.31 (0.62-2.77)	.49
Use of general digital health (yes vs no)		4.41 (2.56-7.60)	<.001	4.33 (2.47-7.61)	<.001	4.19 (2.37-7.41)	<.001
**Barriers to seeking traditional clinical mental health services**							
	Stigma-related barriers (1-4)	1.23 (0.84-1.81)	.29	N/A	N/A	N/A	N/A
	Instrumental or attitudinal barriers (1-4)	1.72 (1.00-2.97)	.05	2.06 (1.11-3.82)	.02	2.05 (1.10-3.80)	.02
Controls, female vs male		1.66 (1.00-2.77)	.05	1.91 (1.08-3.36)	.03	1.88 (1.07-3.23)	.03

^a^Higher scores equal greater amounts. The unadjusted models examined 5 potential additional control variables (mental health need, wellness, perceived stress, socioeconomic status, and geography), and *P*<.20 for none of them; thus, they were excluded from the adjusted models.

^b^Nagelkerke *R*^2^=0.20; *P*<.001.

^c^Nagelkerke *R*^2^=0.20; *P*<.001.

^d^OR: odds ratio.

^e^aOR: adjusted odds ratio.

^f^N/A: not applicable.

## Discussion

### Principal Findings

This study explored the acceptability of using digital health to promote MH among university students in Bangladesh. Although the MH of the sample was comparable with other Bangladeshi university student samples [37,51], with 43.4% (135/311) of the sample experiencing symptoms of depression, we cannot compare the percentage of students who use smartphones with other studies as other studies included smartphone use as an eligibility criterion in research pertaining to the digital field [52,53]. In this sample, nearly all the students owned a personal smartphone (308/311, 99.7%). We found a similar percentage of students (135/311, 43.4%) who self-reported use of digital health, defined as answering *I use digital health services to better my health (excluding use for mental health) currently* in the affirmative, to that of Waldman et al [54], who found that 45% of their Bangladeshi student sample reported looking up health-related information on the internet. To our knowledge, no other studies have examined digital health for MH promotion in Bangladesh; however, our findings show that most students would be likely to use digital health for MH. Our findings also support previous research that shows that those who are more cognizant of their health—in this case, those who already use digital health for general health—are more likely to also be attuned to their psychological health [55]. Specifically, we found that people who use digital health in general have 4 times the odds of also using digital health for MH compared with those who do not use digital health at all.

Overall, the findings partially confirm the hypotheses that the constructs of the TAM (perceived ease of use, usefulness, and social influence) are essential precursors of intention to use and actual use of digital health in general and digital health for MH. We found that the TAM constructs were particularly useful in predicting the intention to use digital health both generally and for MH, as can be seen by the model fit statistics, which included control variables (Nagelkerke *R*^2^=0.445-0.492).

Interestingly, the TAM constructs were predictive of the intention to use both general digital health and digital health for MH but not for the actual use of either (in the adjusted models); this may be because university students in Bangladesh are unaware of or do not have the type of digital health that they would prefer to use. This aligns somewhat with the TAM as the model posits that intention mediates the connection between these constructs and actual use [44]. Therefore, theoretically, ease of use, social influence, and perceived usefulness should be stronger predictors of intention. We also found intention to be a strong predictor of actual use in the unadjusted analysis. These findings suggest that it is necessary for people to think that the use of a product (in this case, a digital health platform) is approved by their social network, easy to use, and valuable for them to form an intent to use the product and that intention is an important precursor to action. This research found that Bangladeshi students would like to use digital health for MH. However, future research should examine whether user-friendly digital health products that promote MH exist for this population to use as previous research shows that usability issues are a barrier to using digital health in general [20].

The results suggest that increasing the use of digital health in general would promote the use of digital health for MH as well. According to our results, one way to do this is to increase the acceptability of digital health products among peer groups as social influence is predictive for general and MH-specific digital health use (and intention toward use). Using user-centered design to ensure that the product is easy to use and meets the users’ needs is also imperative as ease of use and perceived usefulness of an app are associated with intention to use and actual use of general digital health and the intention to use digital health for MH. From an implementation science perspective, this information is critical when developing health communication strategies around health promotion [56]. University administrations can use these findings to encourage transparency regarding health promotion directly, which may indirectly affect the use of digital health for MH.

The results show that university students are open to the use of digital health for MH in Bangladesh and that digital health may be particularly useful for those who may not otherwise seek clinical care as those with higher instrumental and attitudinal barriers are twice as likely to use digital health for MH. We also found that women had higher odds of using digital health for MH, although previous research in rural Bangladesh found that women were less aware of mHealth services than men even though intentions to use mHealth were high regardless of gender [57]. This difference in findings may be because, in this sample, university students had access to mobile phones across genders, unlike in the rural sample, where women had lower rates of phone ownership than men [57]; as such, our findings may show that, when given access, women translate their intention to use mHealth into actual use of such platforms. Previous research in Bangladesh found that women are also less likely to seek physical health care than men [58], although gender differences in MH service use have not been assessed in this population. In other populations where gender differences in MH service use have been researched, it has been found that women seek traditional MH care more than men [59]. In any case, these study results indicate that an MH promotion program on a digital platform may benefit this subgroup of people who may face attitudinal or instrumental barriers to seeking clinical care. This information can be key in marketing as the app can be framed so that it can be used autonomously. To our knowledge, no current app exists that was developed with empirical evidence for a Bangladeshi student population; as such, next steps would entail developing and pilot-testing such an app.

### Limitations

The limitations of this study stem from its cross-sectional design and convenience sample. Owing to the study design, the results were not able to determine causality, nor are they generalizable to the Bangladeshi university student populations at large. As the study’s topic was MH promotion, it is possible that respondents were inclined toward this approach, and those who were not interested in MH promotion did not participate. We cannot conclude that a digital MH promotion program would be of interest to all university students in Bangladesh; however, we can say that this type of program shows promise for students who may not be inclined to receive clinical MH care.

### Conclusions and Future Directions

This study is the first of its kind to examine the acceptability of digital health for MH in Bangladesh. The results show that students are quite open to using digital health as a tool to improve their MH and highlight the influence that social networks might have on this decision-making. As these findings provide evidence of the acceptability of using digital health for MH, future research should pilot-test messaging for a self-paced MH app, such as a meditation app or a web-based intervention, in Bangla. Universities should promote these mental wellness programs to their students.

## Abbreviations

aOR: adjusted odds ratio
MH: mental health
mHealth: mobile health
SES: socioeconomic status
TAM: Technology Acceptance Model
